# Role of AIM in *Corynebacterium*-induced granuloma formation in mice

**DOI:** 10.1186/1476-5926-3-S1-S44

**Published:** 2004-01-14

**Authors:** Kazuhisa Kuwata, Hisami Watanabe, Takashi Yamamoto, Toru Miyazaki, Makoto Naito

**Affiliations:** 1 Department of Cellular Function, Division of Cellular and Molecular Pathology, Niigata University Graduate School of Medical and Dental Sciences, 1 Asahimachi-dori, Niigata 951-8510 Japan; 2 Division of Cellular and Molecular Immunology, Center of Molecular Biosciences, University of the Ryukyus, Nishihara, Okinawa, Japan; 3 Center for Immunology, The University of Texas Southwestern Medical Center, Dallas, Texas, USA

## Introduction

Apoptosis inhibitor expressed by macrophages (AIM) is a murine macrophage-specific protein and belongs to the macrophage scavenger receptor cysteine-rich domain superfamily. AIM has been introduced as the inducer of resistance to thymocyte apoptosis [1]. Because apoptosis of inflammatory cells plays a pivotal role in inflammation [2], we have applied a mouse model to address potential involvement of AIM in the process of granulomatous inflammation *in vivo*.

## Methods

### Animals

Mice deficient in AIM (AIM-/-) were generated by disruption exon 3 of the AIM gene (1). AIM-/- and wild-type (AIM+/+) mice were used. Heat-killed *Corynebacterium parvum *(*C. parvum*), 0.5 mg, was injected into the tail vein. All mice were killed under diethyl ether anesthesia at various time intervals after injection.

### Histology

Formaldehyde-fixed and paraffin embedded livers were sectioned and stained with hematoxylin and eosin for light microscopy.

### Flow Cytometric Analysis and Detection of Apoptosis

The surface phenotype of leukocytes obtained from livers was analyzed using fluorescein isothiocyanate- (FITC), phycoerythrin-, or biotin-conjugated anti-CD3 and anti-NK1.1 monoclonal antibodies in conjunction with a two- or three-color immunofluorescence test. To determine the percentage of cells undergoing apoptosis, FITC-labeled Annexin-V was used.

## Results

**Figure 1:**
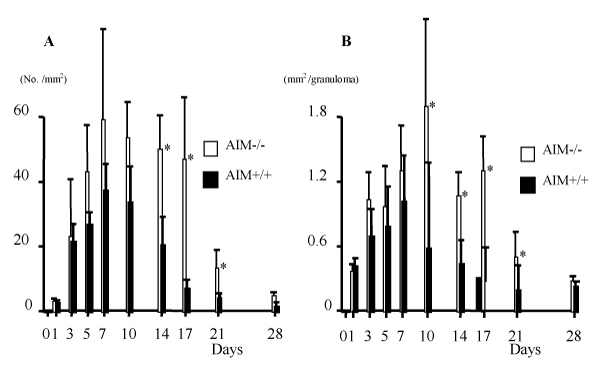
The number of granulomas (A) and area size per granuloma (B) in the livers of AIM-/- and AIM+/+ mice after *C. parvum *injection. AIM-/- mice developed larger numbers of granulomas than AIM+/+ mice. Mean – SD of five mice. *, P &lt; 0.05.

**Figure 2:**
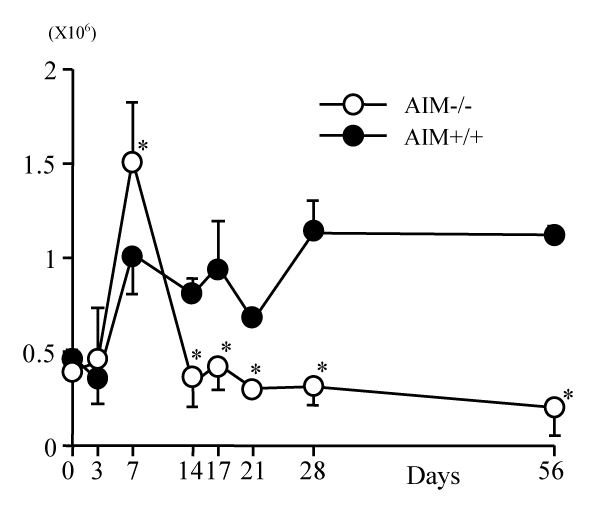
Absolute numbers of NKT cells in the liver of AIM-/- and AIM+/+ mice after *C. parvum *injection. The numbers of NKT cells in AIM-/- mice were significantly smaller than in AIM+/+ mice from day 14 after *C. parvum *injection. Mean – SD of five mice. *, P &lt; 0.05.

The number of granulomas and the area size per granuloma in AIM-/- mice were larger than those in AIM+/+ mice (Figure 1). By flow cytometric analysis, there were numerical increases of conventional T cells, natural killer (NK) and NKT cells after *C. parvum *injection in the liver of both types of mice. After day 7 the numbers of NKT cells in AIM+/+ mice remained at high levels, but there was a rapid decrease of those in AIM-/- mice (Figure 2). By apoptosis detection by Annexin V, larger numbers of intrahepatic NKT cells and conventional T cells underwent apoptosis in the AIM-/- mice than in AIM+/+ mice (data not shown).

## Discussion

**Figure 3:**
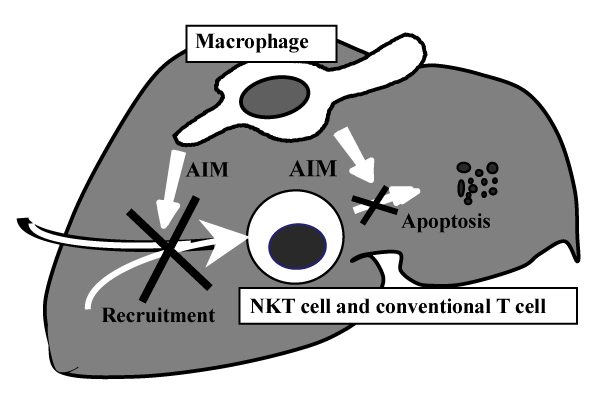
AIM regulates NKT and T cell apoptosis and recruitment for the formation and resorption of hepatic granulomas.

NKT cells play a primary role in the granulomatous response of mice [3] and are associated with resistance to infection against various pathogens [4]. The present study demonstrated the poor repopulation of NKT cells in the middle and late stages of granuloma formation in AIM-/- mice. We have also observed that apoptosis of NKT and T cells after *C. parvum *injection was more prominent in AIM-/- mice than in AIM+/+ mice. These findings suggest that AIM regulates NKT and T cell apoptosis and recruitment and plays an important role in granuloma formation (Figure 3).
